# Endocrine therapy in the years following a diagnosis of breast cancer: A proof of concept study using the primary care prescription database linked to cancer registration data

**DOI:** 10.1016/j.canep.2019.04.012

**Published:** 2019-08

**Authors:** Gabrielle Emanuel, Katherine E. Henson, John Broggio, Jackie Charman, Kieran Horgan, David Dodwell, Sarah C. Darby

**Affiliations:** a National Cancer Registration and Analysis Service, Public Health England, Wellington House – 6th Floor, 133-155 Waterloo Rd, London, SE1 8UG, United Kingdom; b Department of Breast Surgery, Bexley Cancer Centre, St James’s University Hospital, Beckett St, Leeds, LS9 7TF, United Kingdom; c Nuffield Department of Population Health, University of Oxford, Richard Doll Building, Old Road Campus, Oxford, OX3 7LF, United Kingdom

**Keywords:** Breast neoplasm, Therapeutics, Tamoxifen, Aromatase inhibitors, Prescriptions

## Abstract

**Introduction:**

National cancer registration data were linked to the Primary Care Prescription Database (PCPD) in England. The level of endocrine therapy (ET) prescribed in women after a diagnosis of breast cancer was studied.

**Materials and methods:**

Cancer registrations for women diagnosed with breast cancer during 1995–2015, who survived to 31st March 2015, were linked to ET prescriptions issued during April–July 2015.

**Results:**

Among 369 277 survivors of breast cancer diagnosed during 1995–2015, 37% were prescribed ET during April–July 2015. Among women whose breast cancer diagnosis was after 31st July 2010, 81% of those recorded with oestrogen receptor positive (ER+ve) disease were prescribed ET compared with only 6% of those with ER-ve disease. Younger women usually received tamoxifen and older women usually received aromatase inhibitors.

**Discussion:**

The pattern of ET use observed in these data corresponds to that expected. This provides confidence in the potential of the PCPD for epidemiological research.

## Highlights

• Prescribing was as expected from clinical practice.
• 90% received ET prescriptions during the second year after diagnosis.
• Prescribing dropped more than five years after diagnosis.
• The majority of younger women (under 55) received tamoxifen.
• The majority of older women (55+) received AIs.

## 1 Introduction

Most women diagnosed with oestrogen receptor positive (ER+ve) breast cancer in England are prescribed endocrine therapy (ET). ET decreases recurrence and increases survival [1] and is the current standard treatment for patients with ER+ve disease in both early and metastatic breast cancer [2]. Adherence to ET was reported to be between 40% and 70% over a five year treatment period [3,4].

In England ET is commonly prescribed for a duration of five years [5]. It is usually initiated in a hospital setting, but the majority of repeat prescriptions are issued in primary care. It has been possible to link cancer registrations in England with prescriptions issued in primary care using the Clinical Practice Research Datalink (CPRD)[6], but the CPRD covers only around 7% of the population. Furthermore, it includes only patients registered with participating general practices. If a patient moves, further prescriptions may not recorded. This has hampered studies of drugs that are used for long periods of time, such as ET in women with breast cancer [7].

The Primary Care Prescription Database (PCPD) is an administrative database used to calculate reimbursement for prescriptions dispensed by community pharmacies. It contains demographic and drug information categorised according to the British National Formulary (BNF) version 68 [8]. The NHS Business Services Authority (NHSBSA) has partnered with the National Cancer Registration and Analysis Service (NCRAS), allowing cancer registration data to be linked with the PCPD. To assess the epidemiological research potential of the PCPD we have evaluated the level of prescribing of ET and selected other drugs in women with breast cancer in England.

## 2 Materials and methods

Cancer registration is managed by NCRAS and data on all patients diagnosed with cancer in England are collected. Cancer registrations for women diagnosed with breast cancer (ICD-10 code C50) in England between 1st January 1995 and 31st July 2015 with no previous cancer were identified. Women who died before 1st April 2015, those living outside of England and those with a date of death before the prescription was processed were excluded. This is because, in the PCPD, the date of the prescription is the date it was processed by the NHSBSA and in very few cases (0.3% of prescription items in the entire dataset [8]), this is after the patient’s death. We collected information on dates of birth, stage, ER and progesterone receptor (PR) status.

Ages were calculated as of the 1st April 2015 and grouped. We calculated the difference in years between the date of breast cancer diagnosis and the end of the reference period (31st July 2015). The confidence intervals were determined using the Wilson score methodology [9].

The cancer registrations were linked to ET prescriptions in the PCPD during a reference period of April to July 2015 using pseudonymised identifiers derived from NHS number and date of birth. For women recorded with early stage ER+ve disease diagnosed during 2010–2015 the registrations were also linked to prescriptions of the following drugs if they were co-prescribed with ET during the reference period: analgesics, statins, oral hypoglycaemics, and anticoagulents. The same was done for oral bisphosphonates co-prescribed with aromatase inhibitors (AIs). To estimate prescribing in the general population, age-weighted averages of individuals prescribed these drugs were calculated. These averages were weighted by individuals in the PCPD by each year of age and were restricted to those having received any prescription during the reference period, excluding individuals prescribed AIs (for the oral bisphosphonates estimates) and ET (for all other estimates). Sex was not specified as it is not included in the PCPD.

## 3 Results

Among 369 277 survivors of breast cancer diagnosed during the years 1995–2015, 61% had ER unknown; 34% had ER+ve; 5% had ER-ve; and 0.02% had ER borderline disease. The average age of women with ER+ve disease was 64. Overall, 37% of the study population were prescribed ET during the reference period. The commonest drugs were tamoxifen (34% of prescriptions) and AIs (64% of prescriptions). For women recorded with ER+ve disease, 69% were prescribed ET compared with 42% of women with borderline ER, 5% of women with ER-ve disease, and 23% of women with unknown ER status.

**Fig. 1:**
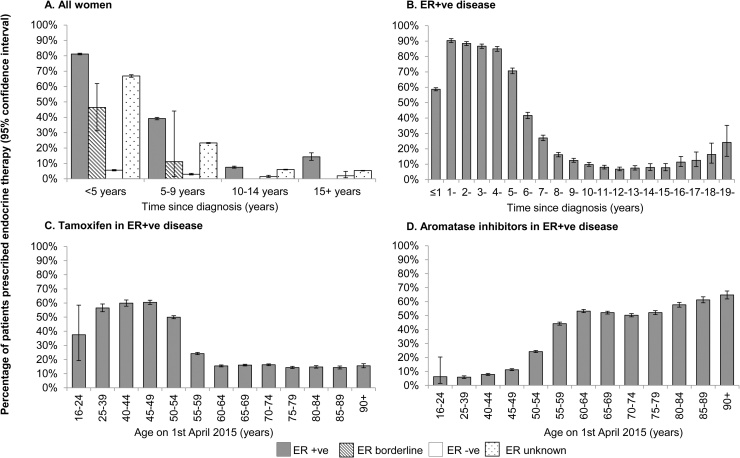
Prescriptions of endocrine therapy during April-July 2015 among women diagnosed with breast cancer in England during 1995–2015. This figure presents the prescribing of endocrine therapy by ER status and time since diagnosis in Panel A. Panel B presents the prescribing of endocrine therapy by time since diagnosis in ER+ve women. Panels C and D present the prescribing of tamoxifen and aromatase inhibitors, respectively, by age. Confidence intervals were determined using the Wilson score methodology.

Among women recorded with ER+ve disease, 81% of those diagnosed after 31st July 2010 (i.e. within the previous five years at the time of the reference period) were prescribed ET compared with 46% of women with borderline ER status and only 6% of women with ER-ve disease (Fig. 1 panel A).

Among women recorded with ER+ve disease 59% of those diagnosed with breast cancer after 31st July 2014 (i.e. during the year prior to the reference period) were prescribed ET as were 90%, 88%, 87% and 85% respectively of women diagnosed during the 2nd, 3^rd^, 4^th^ and 5^th^ years previously (Fig. 1 panel B). For women diagnosed before this, the percentage of women still being prescribed ET during the reference period decreased progressively down to 7% during the 13^th^ year before the reference period, and then increased slowly to 24% in the 20^th^ year before the reference period.

For women recorded as ER+ve and aged less than 55, tamoxifen was the commonest drug and was prescribed to 55% of them (Fig. 1 panel C), while for ER+ve women aged 55 and older AIs were the commonest drug and were prescribed to 52% of them (Fig. 1 panel D).

Among women recorded with ER+ve disease, 22% of women who were prescribed AIs were co-prescribed oral bisphosphonates (3% in the general population). Other drugs commonly co-prescribed with ET to ER+ve women were analgesics (27% compared to 21% in the general population) and statins (24% compared to 24% in the general population). Much smaller percentages of women were co-prescribed oral hypoglycaemics (7% compared to 8% in the general population) or anticoagulents (4% compared to 4% in the general population).

## 4 Discussion

This proof of concept study has demonstrated the ability of the PCPD to identify prescriptions of ET issued in primary care to women in the years following a diagnosis of breast cancer. This is the first such data to become available for the entire population of England.

Most women with early breast cancer will not usually be prescribed ET until their other cancer treatments are completed, which usually takes a few months. Also, the initial ET prescription may be issued in hospital. Therefore, the best estimate of the overall proportion of ER+ve women who are prescribed ET is the percentage of women recorded in the PCPD who received it during the second year after their breast cancer diagnosis. During this period ET prescriptions were issued to 90% of women recorded as ER+ve.

Prescribing of ET decreased after around 5 years after breast cancer diagnosis which was expected as, during the time-period studied, guidelines for ET in early breast cancer recommended that it be prescribed for five years. ET is also recommended for metastatic breast cancer, so it was expected that ET prescriptions would be issued for a percentage of women in the second decade after breast cancer diagnosis, mostly for the treatment of metastatic disease.

A small proportion of ER-ve patients in the cohort were prescribed ET. A quarter of these women were recorded as PR positive, and the prescription of ET is in accord with evidence that tamoxifen increases survival in patients with ER-ve/PR+ve cancers [10].

The choice of ET type is related to menopausal status, with tamoxifen suitable at any age [5] but AIs suitable only for postmenopausal women or (much less commonly), younger women rendered postmenopausal. The patterns of ET prescriptions in the PCPD were in accordance with these scenarios.

The analysis of other drugs co-prescribed with endocrine therapy in ER+ve women with early breast cancer provides insight into the use of oral bisphosphonates in managing AI-associated bone loss [5] and of analgesics for the possible treatment of AI-induced arthralgia [11], while prescriptions of drugs associated with non-breast-cancer comorbidities such as statins and anticoagulents to treat cardiovascular disease and oral hypoglycaemics for diabetes were lower or similar to the general population.

ET has contributed substantially to patient survival [12,13] and prior to the linkage of PCPD to cancer registration data in England, ET prescribing in women with breast cancer could not be reliably captured for the entire population [14,7]. A key strength of the PCPD is its national and near complete coverage [8]. The coverage of the study population by the PCPD was high, with 86% of patients having received a prescription during the reference period. Some patients may have been missed due to not receiving a prescription during the short time frame covered by the reference period. An additional limitation of the PCPD is that age is missing for approximately 20% of prescriptions, potentially impacting the general population estimates for the co-prescribed drugs. Furthermore, as osteoporosis increases with age and women have higher rates of osteoporosis than men [15], our estimate of the prescribing rate of bisphosphonates is likely to underestimate the rate in older women and overestimate the rate in the entire population.

Prescribing was consistent with clinical practice and in accordance with ER status, patient age, and anticipated treatment duration. This study therefore provides confidence in the use of the PCPD for epidemiological purposes. A project is underway within PHE with the intention of making the prescriptions dataset available for request via the Office for Data Release [8] (ODR). This dataset has the potential for many applications, including providing a proxy for multimorbidity, as well as an insight into trends in prescribing both before and after cancer diagnosis and in end of life care. It could also be used to understand geographical differences in prescribing, adherence to guidance and to identify patterns in prescribing which could potentially highlight opportunities for earlier diagnosis. In the future, the PCPD should bridge a substantial gap in the knowledge of therapies that are not hospital based, especially those such as ET where primary care based prescription is the norm and continues over time.

## Ethics approval and consent to participate

The authors declare that ethical approval and patient consent was not applicable.

## Availability of data and material

The authors declare that there is no additional data available in relation to this study.

## Conflict of interest

All authors declare that they have no conflicts of interest in relation to this work.

## Funding

This work was supported by Cancer Research UK [grant number C8225/A21133].

There is no other funding to declare in relation to this work.

## Authors’ contributions

DD, SD and KH designed the study and provided clinical support.

GE conducted the analysis with analytical support from KEH, JB and JC. All authors made substantial contributions to the interpretation of the findings.

All authors contributed to drafting the manuscript or revising it critically for important intellectual content and approved the final version submitted.

## Figures and Tables

**Table A1:** Number of patients prescribed endocrine therapy by ER status during April–July 2015 among women diagnosed with breast cancer in England during 1995–2015.

ER status	Patients prescribed ET	All patients
Positive	85,692	124,240
Negative	976	20,258
Borderline	33	79
Unknown	51,089	224,700
Total	137,790	369,277

**Table A2:** Number of ER+ve patients prescribed endocrine therapy by time since diagnosis during April–July 2015 among women recorded with ER+ve breast cancer in England during 1995–2015.

Time since diagnosis	ER+ve patients prescribed ET	ER+ve patients
≤5 years ago	76,043	93,735
6 to 10 years ago	9,025	23,014
11 to 15 years ago	494	6,580
>15 years ago	130	911

**Table A3:** Number of ER-ve patients prescribed endocrine therapy by time since diagnosis during April–July 2015 among women recorded with ER-ve breast cancer in England during 1995–2015.

Time since diagnosis	ER-ve patients prescribed ET	ER-ve patients
≤5 years ago	842	15,065
6 to 10 years ago	112	3,750
≥11 years ago	22	1,443

**Table A4:** Number of ER unknown patients prescribed endocrine therapy by time since diagnosis during April–July 2015 among women recorded with ER unknown breast cancer in England during 1995–2015.

Time since diagnosis	ER unknown patients prescribed ET	ER unknown patients
≤5 years ago	24,095	36,051
6 to 10 years ago	21,465	92,152
11 to 15 years ago	3,506	58,307
>15 years ago	2,023	38,190

**Table A5:** Number of ER+ve patients prescribed endocrine therapy by individual year since diagnosis during April–July 2015 among women recorded with ER+ve breast cancer in England during 1995–2015.

Years since diagnosis	ER+ve patients prescribed ET	ER+ve patients
≤1	12,844	21,870
1	19,432	21,512
2	17,361	19,633
3	14,967	17,257
4	11,439	13,463
5	5,473	7,746
6	1,599	3,838
7	881	3,257
8	507	3,136
9	341	2,743
10	224	2,294
11	167	2,086
12	131	1,922
13	105	1,404
14	50	638
15	41	530
16	48	421
17	27	216
18	24	147
≥19	31	127

**Table A6:** Number of ER+ve patients prescribed tamoxifen and aromatase inhibitors by age during April–July 2015 among women recorded with ER+ve breast cancer in England during 1995–2015.

Age as of April 2015	ER+ve patients with a tamoxifen prescription	ER+ve patients with an aromatase inhibitor prescription	ER+ve patients
16-39	1,622	169	2,881
40-44	2,836	370	4,734
45-49	6,113	1,133	10,107
50-54	8,139	3,944	16,290
55-59	3,495	6,392	14,467
60-64	2,381	8,197	15,403
65-69	3,152	10,233	19,648
70-74	2,381	7,346	14,623
75-79	1,466	5,340	10,242
80-84	1,157	4,533	7,857
85-89	713	3,055	4,989
90+	465	1,942	2,999

## Appendix

Table A1, Table A2, Table A3, Table A4, Table A5, Table A6
